# Dosimetric evaluation of a simple planning method for improving intensity-modulated radiotherapy for stage III lung cancer

**DOI:** 10.1038/srep23543

**Published:** 2016-03-24

**Authors:** Jia-Yang Lu, Zhu Lin, Jing Zheng, Pei-Xian Lin, Michael Lok-Man Cheung, Bao-Tian Huang

**Affiliations:** 1 Department of Radiation Oncology, Cancer Hospital of Shantou University Medical College, Shantou, Guangdong, China; 2 Department of Laboratory, Shantou Central Hospital, Affiliated Shantou Hospital of Sun Yat-sen University, Shantou, Guangdong, China; 3 Department of Nosocomial Infection Management, the Second Affiliated Hospital of Shantou University Medical College, Shantou, Guangdong, China; 4 Department of Clinical Oncology, Prince of Wales Hospital, Shatin, Hong Kong, China

## Abstract

This study aimed to evaluate the dosimetric outcomes of a base-dose-plan-compensation (BDPC) planning method for improving intensity-modulated radiotherapy (IMRT) for stage III lung cancer. For each of the thirteen included patients, three types of planning methods were applied to obtain clinically acceptable plans: (1) the conventional optimization method (CO); (2) a split-target optimization method (STO), in which the optimization objectives were set higher dose for the target with lung density; (3) the BDPC method, which compensated for the optimization-convergence error by further optimization based on the CO plan. The CO, STO and BDPC methods were then compared regarding conformity index (CI), homogeneity index (HI) of the target, organs at risk (OARs) sparing and monitor units (MUs). The BDPC method provided better HI/CI by 54%/7% on average compared to the CO method and by 38%/3% compared to the STO method. The BDPC method also spared most of the OARs by up to 9%. The average MUs of the CO, STO and BDPC plans were 890, 937 and 1023, respectively. Our results indicated that the BDPC method can effectively improve the dose distribution in IMRT for stage III lung cancer, at the expense of more MUs.

Lung cancer constitutes a major source of mortality in the world and the disease is usually diagnosed in advanced stages^1^,^2^. Concurrent chemoradiotherapy (CCRT) represents the standard of care for patients with stage III non-small cell lung cancer (NSCLC)^3^. Previous studies demonstrated that the use of intensity-modulated radiation therapy (IMRT) improved plan quality, reduced the toxicity, and improved local control and survival rates^4^. However, treatment of larger tumors has been reported to be associated with increased risk of severe radiation pneumonitis and esophageal toxicity^3^,^5^,^6^. Verbakel *et al.*^7^ implemented a hybrid intensity-modulated radiotherapy technique to achieve lung sparing for stage III lung cancer treatment, however, radiotherapy complications such as radiation esophagitis and radiation-induced heart diseases were not alleviated in their method. Therefore, it is essential for us to develop another method to spare the organs at risk (OARs).

On the other hand, because the optimizers of current treatment planning system use simplified algorithms instead of full volume dose algorithms for fast dose computation, the finally calculated dose was subject to an optimization-convergence error (OCE)^8^,^9^, which leads to the discrepancy between the optimizer and finally calculated dose and thus resulting in suboptimal deliverable treatment plan. Especially in lung cancer cases with low density in lung tissue, significantly lower dose in the target containing lung tissue^10^ and a heterogeneous dose distribution can be observed in the deliverable treatment plan. The OCE is a systematic error, thus it could not be overcome by designing the optimal beam arrangement and number, although this approach is usually effective for improving IMRT plan quality^11^,^12^.

In this study, we proposed a base-dose-plan-compensation (BDPC) planning method to improve the IMRT plan quality for stage III lung cancer patients, by means of compensating for the OCE utilizing a base dose plan (BDP). To evaluate the efficacy of the introduced planning method, two other methods were used as references for comparisons.

## Methods

### Ethics Statement

The protocol was approved by the Ethical Commission of the Cancer Hospital of Shantou University Medical College. Because this was not a treatment-based study, our institutional review board waived the need for written informed consent from the participants. The patient information was anonymized and de-identified to protect patient confidentiality. The methods were carried out in accordance with the approved guidelines.

### Patient characteristics

From February 2014 to November 2014, thirteen patients suffering from primary NSCLC in the Cancer Hospital of Shantou University Medical College were included in this study. Basic characteristics of the patients were summarized in Table 1. Staging was according to the American Joint Committee on Cancer (AJCC) 7^th^ edition.

### CT simulation

All patients were scanned in supine position with the arms above their heads. A vacuum bag (Medtec Medical, Inc, Buffalo Grove, IL) was used to immobilize the thoracic regions. All the patients received a contrast-enhanced scan with a Big Bore Brilliance CT (Philips Medical Systems, Inc., Cleveland, OH, United States). CT Images were acquired at a 5 mm slice thickness during normal breathing. The CT images were then transferred to the Eclipse version 10.0 treatment planning system (Varian Medical System, Inc., Palo Alto, CA) for target and OAR delineation.

### Target and OAR delineation

The gross tumor volume (GTV) was defined as the primary tumor displayed at lung window and the clinically positive lymph nodes seen on the enhanced CT or positron emission tomography (PET). The clinical target volume (CTV) was expanded by the GTV with variable 5–10 mm margin, which was the combination of high risk of microscopic tumor extension and variable tumor motion different from patient to patient. The planning target volume (PTV) was defined by adding a 5 mm margin in the axial direction and 1 cm in the superior-inferior direction to the CTV to account for patient positioning uncertainty and mechanical tolerance. The OARs included the contralateral lung, ipsilateral lung, spinal cord, esophagus, heart and normal tissue (NT, defined as the body minus PTV). The mean PTV volume was 275 cm^3^ (ranged from 205–409 cm^3^). All the OAR contouring were according to the RTOG 1306 criterion^13^.

### IMRT planning

Generally, six coplanar 6 MV photon fields from a Truebeam (Varian Medical System, Inc., Palo Alto, CA) accelerator were created for each plan in Eclipse. The beam angles were set at 330°, 20°, 70°, 120°, 165° and 210° when the tumor was located at the left lung. When the tumor was located at the right lung, the beam angles were set at 195°, 240°, 290°, 340°, 30° and 150°. The beam arrangements were set according to Lievens’s study^14^. Several ring-like structures were contoured in order to make the isodose lines more conformal to the target volume. The prescription was set to 2 Gy × 30 fractions. The Dose Volume Optimizer (DVO, version 10.0.28) algorithm was used for plan optimization. When the value of the objective function approached a minimum and showed no further decrease, a smart leaf motion calculator (SLMC) algorithm was used to calculate the multi-leaf collimator (MLC) motion. The final dose calculation was performed using Anisotropic Analytical Algorithm (AAA, version 10.0.28) with a grid size of 2.5 mm. The treatment plan was normalized to ensure that 90% of the PTV was covered by the prescription.

In the optimization objectives settings, PTV coverage was assigned the highest priority, followed by the avoidance of excessive dose to the OARs. Detailed dose constraints^7^,^13^ were listed in Table 2. For the conventional optimization (CO) method, the objectives were adjusted whenever necessary to make the plan clinically acceptable. For a split-target optimization (STO) method, the PTV was divided into two components: the PTV_soft, with a density in the soft-tissue range, and the PTV_lung with a density in the lung range. The optimization objective was set to 2 to 4 Gy higher for PTV_lung and other objective were set the same as the CO plan. For the BDPC method, we utilized the “base dose plan” function incorporated in Eclipse, which can enable the treatment planning system to optimize a plan, as a “top dose plan (TDP)”, while taking another plan (as a BDP) into consideration during the optimization process, with the aim of achieving optimal plan sum by making up for inadequacies (hot and cold spots) in the BDP. The BDPC procedure is described as follows: (1) the number of fractions of the CO plan was modified to a half of prescribed number of fractions (from 30 to 15 fractions in our cases) to generate a BDP with a half of the total prescribed dose (30 Gy); (2) the BDP was duplicated to generate a TDP (30 Gy); (3) keeping the optimization objectives unchanged, the TDP was further optimized based on the BDP using Eclipse’s “base dose plan” function with 20 maximum iterations (at this point, the prescribed dose of the plan sum of TDP and BDP was equivalent to the originally prescribed dose of 60 Gy); (4) the final dose of the optimized TDP (30 Gy) was calculated; (5) the number of fractions of the optimized TDP was changed from a half (15 fractions) to the total prescribed number of fractions (30 fractions), leading to that the prescribed dose of the top dose plan was restored from a half (30 Gy) to the total prescribed dose (60 Gy); (6) the final TDP with the prescribed number of fractions was referred to as the BDPC plan.

### Plan evaluation

D_98%_, D_2%_, D_50%_, conformity index (CI) and homogeneity index (HI) was evaluated for PTV among the three planning methods. D_x%_ represents the dose received by x% volume of the organ. For example, D_50%_ means the dose received by 50% volume of the organ. The CI proposed by Paddick^15^ was defined as the location of the prescription isodose volume (PIV) with respect to the target volume (TV). HI is defined by the following formula according to the recommendations of ICRU report 83^16^. The CI value was between 0 and 1 with 1 representing ideal conformity. On the contrary, the HI value of 0 represented ideal homogeneity in the target.


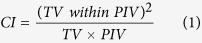



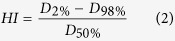


The maximum dose and various dose-volume parameters to specific OARs were generated for the plans to assess their effectiveness in OAR sparing. V_x_ stands for the volume of the organ receiving a dose of ≥x Gy. For example, V_40_ means the volume of organ receiving a dose of ≥40 Gy. Specifically, the spinal cord was assessed in terms of its maximum dose. The total lung (T-L) was evaluated using V_5_, V_10_, V_20_, V_30_ and the mean lung dose (MLD); the contralateral lung (C-L) was evaluated using V_5_; the esophagus was appraised with the maximum dose, mean dose, V_35_, V_50_ and V_60_. The heart was assessed in terms of V_30_, V_40_ and mean dose. Monitor units (MUs) per fraction were compared among the three planning methods.

### Statistical analysis

Data analysis was carried out using the SPSS version 19.0 software (SPSS, Inc., Chicago, IL, USA). The differences among the BDPC, STO and CO plans were evaluated using repeated measures ANOVA. When *p* of <0.05 was achieved, a further Least Significant Difference (LSD) measurement was performed to compare the difference between groups. Differences were considered to be statistically significant when *p* was <0.017 due to the adjustment of the observed significance level by one third.

## Results

### Target coverage and MUs

**Figure 1:**
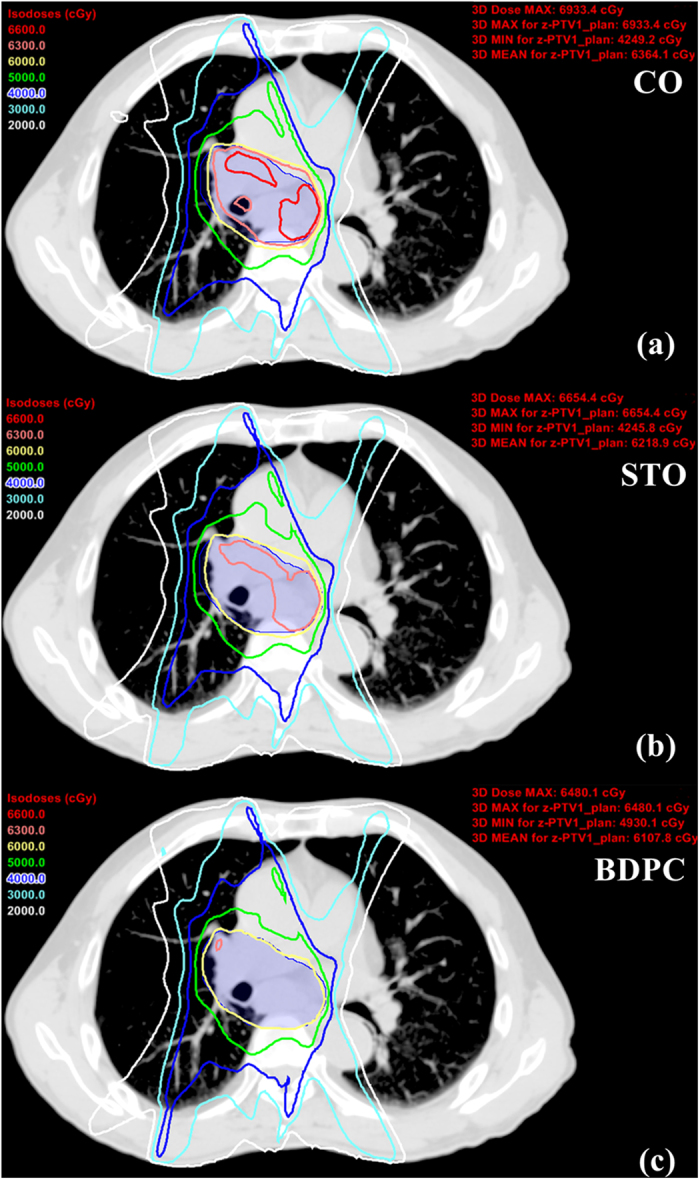
Isodose distribution for the conventional optimization (CO) (**a**), split-target optimization (STO) (**b**) and base-dose-plan-compensation (BDPC) planning methods (**c**) from one representative case.

**Figure 2:**
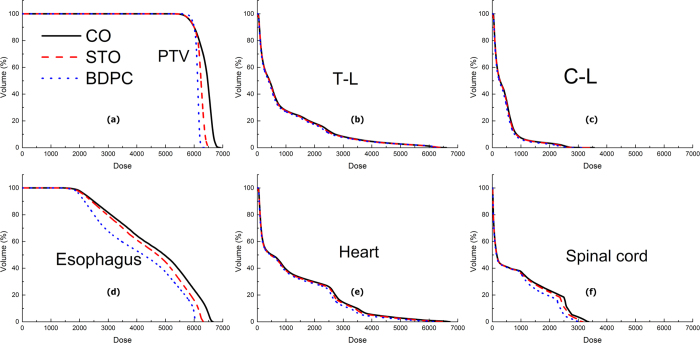
Dose-volume histograms (DVHs) of the planning target volume (PTV) (**a**) and organs at risk (OARs) (**b–f**) for the conventional optimization (CO), split-target optimization (STO) and base-dose-plan-compensation (BDPC) planning methods. T-L = total lung; C-L = contralateral lung.

All the plans created by the three planning methods fulfilled the specified dose constraints. The BDPC method achieved more homogeneous dose distribution, irrespective of isodose distribution (Fig. 1) and dose volume histogram (DVH) display (Fig. 2a). Table 3 summarized the dose coverage parameters and MUs among the three planning methods. It could be seen from Table 3 that the BDPC method obtained significantly higher D_98%_ (9.7 ± 0.9% and 1.3 ± 0.9% higher than the CO and STO) of the PTV. Meanwhile, it also achieved lower D_2%_ (5.8 ± 1.7% and 2.7 ± 0.9% lower than the CO and STO). With regard to the HI, BDPC was significantly superior to CO and STO by 54.4 ± 8.9% and 37.6 ± 9.6%, respectively. With regard to the CI, BDPC was better than CO and STO by 6.9 ± 3.8% and 3.4 ± 2.1%, respectively. However, BDPC required more MUs than the other two methods. The MUs were increased by 15.0 ± 3.2% and 9.3 ± 3.9% compared to CO and STO, respectively.

### OARs sparing

All the OARs exhibited lower dose with the BDPC method. The numerical statistics from the DVH analysis were listed in Table 4. The V_35_, V_50_, V_60_ and mean dose to the esophagus were reduced by 3.2%, 3.0%, 9.1% and 7.0% compared to CO, and by 2.5%, 1.9%, 5.6%, 4.5% compared to STO. The V_5_, V_10_, V_20_, V_30_ and MLD of the T-L were reduced by up to 2.2%. The V_5_ of the C-L was 4.0% and 3.4% lower compared to CO and STO, respectively. BDPC also reduced the V_30_, V_40_ and mean dose of the heart by 1.8%, 1.1% and 4.4% compared to CO and by 1.0%, 0.7% and 2.1% compared to STO, and it reduced the spinal cord dose by 3.1% compared to CO. Additionally, BDPC resulted in the least volume receiving a high dose (107% of the prescribed dose) in the normal tissue. Figure 2b–f displayed the DVH of the OARs for the three planning methods in one representative case. It appeared that most of the DVH curves of the OARs of BDPC shifted to the left, indicating lower dose received.

## Discussion

To improve the therapeutic ratio and obtain optimal clinical outcomes, it is essential to improve the planning technique to give full scope to the advantages of IMRT for lung cancer, that is, to achieve better target dose homogeneity, conformity and better OARs sparing. Our study demonstrated that the introduced BDPC planning method has the ability to further improve the IMRT for stage III lung cancer.

The main advantage of the BDPC method lies in its homogeneous dose distribution in the target with significantly fewer hot and cold spots with an improvement by 38–54%. For lung radiotherapy treatment, PTV was typically generated to account for position, size, and shape caused by respiratory motion and uncertainties during patient positioning and alignment of the therapeutic beams during the treatment^17^. Underdosage in the PTV may result in insufficient dose to the tumor and may lead to the likelihood of tumor recurrence^18^, because the tumor control probability (TCP) predominately correlates with the minimum dose of tumor^19^. And overdosage may result in severe acute reactions in tissues (such as esophagus) or late complications^20^. Accordingly, the improvement of homogeneity may have potential clinical benefits of lowering the risk of tumor recurrence and decreasing the radiation-induced toxicity due to unnecessary excessive dose.

Furthermore, the BDPC method provided superior conformity by 3.4–6.9%, which could better spare the surrounding normal tissue. All the OARs exhibited 0.7–9.1% dose reduction with the proposed BDPC method. The findings are attractive because a number of studies have reported the association between the dose-volume predictors and the incidence of complications in lung radiotherapy treatments. The common complications include the radiation-induced pneumonitis, radiation esophagitis and radiation-induced heart diseases^21^,^22^,^23^. Many dosimetric predictors, such as V_5_, V_10_, V_20_, V_30_ and MLD of T-L were reported to be associated with radiation-induced pneumonitis^21^,^24^,^25^,^26^,^27^,^28^. Song *et al.*^29^ found correlations between fatal pneumonitis and CL-V_5_ dose and he suggested that CL-V_5_ should be kept to be less than 60%. Radiation esophagitis was another common complication experienced by lung cancer patients receiving radiotherapy treatment^30^. These complications significantly affected quality-of-life and could negatively impact long term survival. Rose *et al.*^6^ related V_35_ and V_60_ to clinically significant radiation esophagitis. Palma *et al.*^31^ determined that the esophageal volume receiving ≥60 Gy (V_60_) alone emerged as the best predictor of grade ≥2 and grade ≥3 radiation esophagitis in patients undergoing concurrent chemoradiation therapy. V_50_^32^,^33^,^34^ and the mean dose^35^ were also associated with the risk of esophageal toxicity. Radiation-induced heart disease had been well documented and was believed to occur during radiotherapy. In a study by Veinot *et al.*^22^, patients who received thoracic radiotherapy showed moderate to significant myocardial fibrosis with heart exposure >30 Gy. The heart V_30_ was reported as a significant predictor^36^ of radiation-induced pericardial effusion, with a V_30_ of >46% associated with a 73% rate of pericardial effusion compared with 13% for a V_30_ of <46%. As all the predictors mentioned above could be further reduced by the BPDC method, the risks of radiation-induced complications may be decreased and the quality of life may be potentially improved for the lung cancer patients.

Traditionally, the “base dose plan” function is usually applied for optimizing a second-course treatment plan (such as a boost plan), while considering the first-course plan, in order to achieve an optimal plan sum (TDP plus BDP) in the optimizer. But the “base dose plan” function is utilized in a different way in our method, because it is used for obtaining a deliverable treatment plan (TDP) with finally calculated dose, not a plan sum in the optimizer. In principle, the “base dose plan” function is adopted to compensate for the OCE. When an OCE introduces a cold spot into the finally calculated dose distribution in the CO plan (BDP), the BDPC plan (TDP) will produce a hot spot in the corresponding location to even out the original cold spot for a uniform summed dose. After calculating the final dose of the optimized BDPC plan (TDP), the OCE introduces a cold spot into the hot-spot region of the BDPC plan (TDP), and finally, the BDPC plan can achieve a uniform dose.

The OCE primarily originated from three major sources including tissue heterogeneity, MLC leaf motion calculation and the optimization algorithm^9^. Possible solutions to the OCE were investigated in several studies. The STO method^7^ is an effective approach to minimize the error arising from tissue heterogeneity, but the errors from the other two sources could not be reduced, so it is not effective enough. By contrast, the BDPC method is able to reduce the overall OCE by compensating for the whole plan and is effective enough. Another planning method proposed by Süss *et al.*^37^,^38^ corrects the OCE by re-optimization with additional optimization objectives to address hot and cold spots, but it is only locally effective and new hot and cold spots may appear in other region. By contrast, the BDPC method is globally effective throughout the entire treatment region. The Direct Aperture Optimization (DAO) technique^39^,^40^,^41^ incorporates the deliverable MLC apertures series instead of ideal fluences in the optimizer to eliminate the error arising from the MLC leaf motion calculation. Unfortunately, this technique is not available in the treatment planning systems without DAO, such as Eclipse version 10.0, whereas the BDPC method is commonly available because a “base dose plan” or similar function is a basic feature for treatment planning systems. Zacarias and Mill^8^ also adopted the “base dose plan” function to overcome the OCE, but that method is not the same as ours, because it required a complex process and additional softwares thus leading to significantly increased planning steps and time. On the contrary, our method is much simpler and practical for routine use, as the only required procedure is modifying one parameter (number of fractions of BDP) and the excellent homogeneous dose distribution can be effortlessly achieved through a single further optimization.

However, the introduced method slightly increased the number of MUs, which was reported to be associated with more treatment time and peripheral dose outside the treatment field, leading to a likelihood of intrafraction shifts of tumor position and radiation-induced secondary cancer^42^,^43^,^44^,^45^. As shown in Table 3, the MUs were 1023 ± 159, 890 ± 134 and 937 ± 145 for the BDPC, CO and STO method, respectively. We infer that the treatment time with the BDPC method is increased by 13.3 and 8.6 seconds on average compared to the CO and STO methods with the dose rate of 600 MU/min. As to the peripheral dose, we find it is increased by 0.32 cGy and 0.21 cGy on average compared to the CO and STO methods (1 MU generates 2.44 × 10^−3^ cGy peripheral dose at 20 cm away from the isocenter according to the results of our previous measurement). Whether these drawbacks will impact on the clinical treatment needs further investigations.

## Conclusions

In this study, we evaluated the dosimetric characteristics of an IMRT planning method, the BDPC method, applied in stage III lung cancer. We found that this method not only improved the conformity and homogeneity of the target but also spared the OARs, thus may increasing the therapeutic ratio. Furthermore, it is simple and effective for routine use. Therefore, the proposed method is recommended for the treatment of stage III lung cancer.

## Additional Information

**How to cite this article**: Lu, J.-Y. *et al.* Dosimetric evaluation of a simple planning method for improving intensity-modulated radiotherapy for stage III lung cancer. *Sci. Rep.* **6**, 23543; doi: 10.1038/srep23543 (2016).

## Figures and Tables

**Table 1:** Basic characteristics of 13 patients.

Characteristic	number
Histology	
Adenocarcinoma	4
Squamous cell carcinoma	9
Central or peripheral	
Central	11
Peripheral	2
Left or right sided	
Left sided	5
Right sided	8
Size of primary tumor and nodal involvement	
T2N2M0	12
T3N1M0	1
Location of primary tumor	
Right middle lobe	3
Left lower lobe	2
Right lower lobe	4
Left middle lobe	3
Right upper lobe	1
Gender	
Male	11
Female	2

**Table 2:** Summary of the dose constraints for stage III lung cancer.

Parameter	Constraint
PTV D_min_	≥90% of prescribed dose
Total body V_110%_	the areas exceeding 110% of the prescribed dose are confined within the PTV
Spinal cord D_max_	<45 Gy
T-L V_20_	<30%
C-L V_5_	<50%
MLD	<20 Gy
Esophagus D_max_	<105% of prescribed dose

PTV = planning target volume; T-L = total lung; C-L = contralateral lung; MLD = mean lung dose; V_x_ = percentage of volume receiving a dose of ≥ x Gy; D_min_ = minimum dose; D_max_ = maximum dose.

**Table 3:** Summary of the target dose coverage parameters and monitor units (MUs) for the three planning methods.

Parameter	BDPC	CO	STO	F	*p*	*p*^1^	*p*^2^
D_2%_ (Gy)	62.46 ± 0.43	66.35 ± 1.10	64.21 ± 0.46	124.62	0.000	0.000	0.000
D_98%_ (Gy)	58.54 ± 0.14	57.34 ± 0.36	57.80 ± 0.49	52.22	0.000	0.000	0.000
D_50%_ (Gy)	61.21 ± 0.18	63.13 ± 0.79	61.92 ± 0.45	64.80	0.000	0.000	0.000
CI	0.88 ± 0.02	0.85 ± 0.02	0.82 ± 0.03	44.39	0.000	0.000	0.000
HI	0.06 ± 0.01	0.14 ± 0.02	0.10 ± 0.01	158.66	0.000	0.000	0.000
MUs	1023 ± 159	890 ± 134	937 ± 145	77.87	0.000	0.000	0.000

Data presented as mean ± standard deviation. D_x_ = the dose received by x of the volume; CI = conformity index; HI = homogeneity index. *p*^1^: BDPC *vs* CO; *p*^2^: BDPC *vs* STO.

**Table 4:** Summary of the dose to organs at risk (OARs) for the three planning methods.

OARs	Parameters	BDPC	CO	STO	F	*p*	*p*^1^	*p*^2^
T-L	V_5_ (%)	53.0 ± 8.8	55.1 ± 8.4	54.7 ± 8.8	26.80	0.000	0.000	0.000
	V_10_ (%)	33.5 ± 6.2	34.2 ± 6.1	34.2 ± 6.3	17.21	0.000	0.000	0.000
	V_20_ (%)	23.5 ± 4.1	24.0 ± 3.9	23.8 ± 4.1	12.98	0.000	0.002	0.000
	V_30_ (%)	14.5 ± 3.9	14.8 ± 3.7	14.6 ± 3.9	12.14	0.000	0.001	0.271
	MLD (Gy)	12.57 ± 2.16	12.79 ± 2.06	12.74 ± 2.15	12.51	0.000	0.001	0.001
C-L	V_5_ (%)	38.2 ± 8.4	42.3 ± 8.6	41.6 ± 8.9	31.97	0.000	0.000	0.000
Esophagus	D_max_ (Gy)	60.92 ± 2.55	64.75 ± 3.72	62.64 ± 3.69	54.67	0.000	0.000	0.001
	V_35_ (%)	36.0 ± 14.0	39.2 ± 15.5	38.5 ± 15.2	9.30	0.008	0.010	0.009
	V_50_ (%)	24.8 ± 12.7	27.8 ± 14.1	26.7 ± 13.6	12.59	0.003	0.003	0.005
	V_60_ (%)	9.8 ± 7.7	18.9 ± 10.9	15.3 ± 8.9	26.97	0.000	0.000	0.001
	D_mean_ (Gy)	25.68 ± 7.25	27.67 ± 8.08	26.90 ± 7.76	30.14	0.000	0.000	0.000
Heart	V_30_ (%)	26.5 ± 15.3	28.3 ± 15.6	27.5 ± 15.3	10.04	0.006	0.007	0.012
	V_40_ (%)	13.1 ± 8.9	14.2 ± 9.1	13.8 ± 8.9	11.24	0.000	0.003	0.009
	D_mean_ (Gy)	17.72 ± 8.24	18.44 ± 8.34	18.12 ± 8.32	24.99	0.000	0.000	0.000
Spinal cord	D_max_ (Gy)	38.85 ± 4.96	40.09 ± 4.81	39.45 ± 4.31	8.64	0.001	0.001	0.069
NT	V_107%_ (cm^3^)	0.0 ± 0.0	1.3 ± 1.8	0.1 ± 0.2	7.51	0.017	0.018	0.173

Data presented as mean ± standard deviation. OARs = organs at risk; T-L = total lung; C-L = contralateral lung; NT = normal tissue; MLD = mean lung dose; V_x_ = percentage of volume receiving ≥ x Gy. D_max_ = maximum dose; D_mean_ = mean dose; V_107%_ = the volume receiving ≥ 107% of the prescription. p1: BDPC vs CO; p2 BDPC vs STO.
